# Frequent EGFR Positivity and Overexpression in High-Grade Areas of Human MPNSTs

**DOI:** 10.1155/2008/849156

**Published:** 2008-08-28

**Authors:** Séverine Tabone-Eglinger, Radislav Bahleda, Jean-François Côté, Philippe Terrier, Dominique Vidaud, Anne Cayre, Alain Beauchet, Nathalie Théou-Anton, Marie-José Terrier-Lacombe, Antoinette Lemoine, Frédérique Penault-Llorca, Axel Le Cesne, Jean-François Emile

**Affiliations:** ^1^INSERM U602, Hôpital Paul Brousse, 12 Avenue P. Vaillant Couturier, 94800 Villejuif, France; ^2^INSERM U590, Centre Léon Bérard, 28 rue Laënnec, 69373 Lyon, Cedex 8, France; ^3^INSERM UMR-S775, Université Paris Descartes, Centre Universitaire des Saints-Pères, 45 r des Saints-Pères, 75006 Paris, France; ^4^Institut Gustave-Roussy, 39 rue Camille Desmoulins, 94805 Villejuif Cedex, France; ^5^INSERM U745, Faculté des Sciences Pharmaceutiques et Biologiques, Université René Descartes, 4 Avenue de L'Observatoire, 75270 Paris, Cedex 6, France; ^6^Anatomie et Cytologie Pathologiques, Centre Jean Perrin, 39 rue Montalembert, 63000 Clermont-Ferrand, France; ^7^Hôpital Ambroise Paré, APHP and Faculté de médecine PIFO, UVSQ - 9, Avenue Charles de Gaulle, 92104 Boulogne, France

## Abstract

Malignant peripheral nerve sheath tumours (MPNSTs) are highly malignant and resistant. Transformation might implicate up regulation of epidermal growth factor receptor (EGFR). Fifty-two MPNST samples were studied for EGFR, Ki-67, p53, and survivin expression by immunohistochemistry and for *EGFR* amplification by in situ hybridization. Results were correlated with clinical data. *EGFR* RNA was also quantified by RT-PCR in 20 other MPNSTs and 14 dermal neurofibromas. Half of the patients had a neurofibromatosis type 1 (NF1). EGFR expression, detected in 86% of MPNSTs, was more frequent in NF1 specimens and closely associated with high-grade and p53-positive areas. MPNSTs expressed more *EGFR* transcripts than neurofibromas. No amplification of *EGFR* locus was observed. NF1 status was the only prognostic factor in multivariate analysis, with median survivals of 18 and 43 months for patients with or without NF1. Finally, EGFR might become a new target for MPNSTs treatment, especially in NF1-associated MPNSTs.

## 1. INTRODUCTION

Malignant peripheral nerve sheath tumours (MPNSTs) are Schwann cell neoplasms that are highly aggressive, frequently lethal, and generally resistant to conventional radiation and chemotherapy [1, 2].

Nearly half of these tumours arise in the context of the inherited predisposition syndrome, neurofibromatosis type 1 (NF1), suggesting that inactivation of the *NF1* tumour suppressor gene might be causally related to the development of these cancers [3]. NF1 is a dominantly inherited human disease affecting one in 2500 to 3500 individuals [4]. NF1 is characterized by café-au-lait spots (flat pigmented skin lesions), Lish nodules (abnormality of the iris), skeletal abnormalities, learning disabilities, neurofibromas, and increased risk of developing malignant tumours of the central and peripheral nervous system [5]. NF1 is associated with mutations of the tumour suppressor gene *NF1*, which encodes for the Ras-GTPase-activating protein neurofibromin [6–8].

Molecular events contributing to peripheral nerve tumour development are unclear. In the context of NF1, loss of neurofibromin, the NF1 protein product, is believed to be the earliest event, as patients inherit a mutated *NF1* allele and lose the second copy in the MPNST cells. Loss of both copies was also observed in benign neurofibromas. It is likely that tumour suppressor mutations alone are not sufficient, and that deregulation and/or mutations of oncogenes are necessary to induce malignant transformation of Schwann cells. The overexpression or mutation of the tumour suppressor gene *TP53* observed in MPNSTs supports the notion that p53 alterations play a role in their development [9]. Several studies have demonstrated the central role of epidermal growth factor receptor (EGFR) in malignant transformation of Schwann cells [10–13]. To our knowledge, only 12 cases of human MPNST have been studied for EGFR by immunohistochemistry [10, 13]. In the present study, we analyzed the expression of EGFR in the tumours of 52 patients with MPNST, and compared it with NF1 status and survival.

## 2. MATERIALS AND METHODS

### 2.1. Patients and samples

Patients of the main series (*n* = 52) were all treated in the Institut Gustave Roussy (IGR, Villejuif, France) between 1985 and 2005. Clinical records were reviewed by one of us (R. Bahleda), with special attention to initial localization, NF1 status, treatment and survival. Diagnosis of NF1 was established according to the NIH criteria [14]. Most of the patients had undergone surgery in another centre and were secondary referred to IGR. Tumours were considered as local stage, when R0 surgery was performed initially, and locally advanced stage for R1 and R2 surgery. Only cases with paraffin embedded MPNST samples were included in the study. Histological review was realized for all included patients by at least two pathologists (PT, MJTL, JFE) on hematoxylin-eosin stained slides. Diagnosis of MPNST was performed according to WHO criteria [15]. Grading of the tumours was not performed, due to limited amounts of paraffin embedded samples. Immunostaining with S100 protein (rabbit polyclonal, Dako, Carpenteria, Calif, USA) and KIT (rabbit polyclonal, Dako) was performed when necessary to confirm diagnosis.

All 52 paraffin embedded samples were subjected to immunohistochemistry; 8 of which were also analysed by FISH/CISH.

Frozen samples from 20 other patients with MPNST were used for the RNA analysis. Sixteen were from a previously published series [16] and four from Léon Bérard Centre (Lyon, France). Frozen control samples from 14 patients with benign dermal neurofibromas were also analyzed.

All samples were obtained from surgery performed for diagnostic and/or therapeutic purpose, and were used according to French ethical regulations.

### 2.2. Immunohistochemistry

Immunohistochemistry was performed on four micron sections from paraffin embedded tumour samples, after antigen retrieval by heating at 95°C for 20 minutes in 10 mM citrate buffer pH6. For mouse monoclonal anti-EGFR (31G7, Zymed, South San Francisco, Calif, USA, final dilution 1/10), P53 (DO-7, Novocastra, Newcastle upon Tyne, UK, final dilution 1/50), and anti-Ki-67 (Mib1 Dako, final dilution 1/50), staining was revealed with LSAB kit (Dako). For anti-Survivin (12C4, Dako, final dilution 1/100) staining was revealed with CSAII (Dako), according to manufacturer's instruction.

For EGFR staining, tumour cells were considered negative, when positive signals were detected on nontumour cells (usually spindle cells and/or small nerves in the periphery of the tumours); otherwise, staining was considered as not interpretable.

### 2.3. Fluorescent in situ hybridization (FISH)

Eight paraffin embedded samples of the main series were analyzed for EGFR amplification. EGFR specific sequence probe (LSI EGFR) and control chromosome enumeration probe 7 (CEP7) were used according to the manufacturers' recommended protocol (Vysis-Abbott Molecular Diagnostics, Baar, Switzerland), but with some minor modifications. The DNA probes and the sections of tissues were denatured at 85°C for 5 minutes using a HYBrite instrument. An additional wash in distilled water was added before counterstaining and mounting with a solution of 4, 6-diamidino-2-phenylindole (DAPI). The results are reported as the ratio of average EGFR/CEP7 signals per nucleus. Signal ratios of <2 were classified as nonamplified (NA) and ≥2 as amplified (A). In each section, at least 30 nuclei were counted for signals.

### 2.4. Chromogenic in situ hybridization (CISH)

CISH experiments were performed, according to the protocol given by the supplier (Zymed), along with FISH to have more information about sample morphology and to have a permanent signal. Results were interpreted as indicated above for FISH.

### 2.5. Real-time PCR

The theoretical and practical aspects of real-time quantitative RT-PCR using the ABI Prism 7700 sequence detection system (Applied Biosystems, Foster City, Calif, USA) have been described in detail elsewhere [16].

The precise amount of total RNA added to each reaction mix (based on optical density) and its quality (i.e., lack of extensive degradation) are both difficult to assess. We therefore also quantified transcripts of the endogenous RNA control gene *TBP* (Genbank accession NM_003194), which encodes the TATA box-binding protein. Each sample was normalized on the basis of its *TBP* content. Results, expressed as N-fold differences in target gene expression relative to the *TBP* gene, and termed “Ntarget,” were determined as Ntarget = 2Δ^Ctsample^, where the Δ^Ct^ value of the sample was determined by subtracting the average Ct value of the target gene from the average Ct value of the *TBP* gene.

The N*target* values of the samples were subsequently normalized such that the mean of the dermal neurofibroma N*target* values was 1.

### 2.6. Statistical analysis

Quantitative data were expressed as mean ± standard deviation; qualitative data as frequency and percent. Comparisons of means were performed using Student's *t*-test or Mann and Whitney nonparametric test when necessary. Comparisons of frequencies were performed using the Chi square test, or Fisher's exact test when necessary.

Log-rank tests were used to examine the relationship between overall survival and the following variables: age, gender, initial localization, NF1 status, and EGFR expression. Variables with a statistical *P* value <.20 were entered into a Cox model multivariate analysis. *P* value less than 0.05 was considered significant in multivariate analysis.

Statistical analyses were performed with the SAS 8.2 software package (SAS Institute Inc, Cary, NC, USA).

## 3. RESULTS

The mains clinical characteristics of the 52 patients with MPNST are presented in Table 1. The mean age at time of diagnosis was 23 ± 15 years, and the sex ratio was 30 m/22 f. Tumours were localized in trunk, head or neck (*n* = 24), or in the limbs (*n* = 28). Half of the patients (*n* = 26) had a NF1, of whom nine had a familial history of NF1. The age at diagnosis of MPNSTs was earlier in patients with NF1 (19 ± 9 years) than in non-NF1 patients (27 ± 18 years) (*P* = .04).

**Figure 1:**
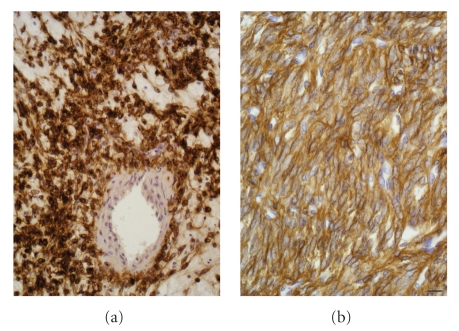
EGFR expression in MPNSTs. In both cases, 100% of tumour cells strongly expressed EGFR (brown). However, it was detected either (a) within the cytoplasm or (b) on plasma membrane. Cell nuclei were stained in blue by hematoxylin. Scale bar represent 20 *μ*m.

EGFR expression by tumour cells was detected by immunohistochemistry in 36 out of the 42 (86%) valuable patients with MPNST; percentages were higher in the NF1 subgroup (95% versus 75%; *P* = .06; Chi square test) (see Table 1). Localization of EGFR within tumour cells was either membranous, cytoplasmic, or both (see Figure 1). In six cases, tumour cells were negative and 10 other cases were not valuable and were thus excluded from the analysis.

**Figure 2:**
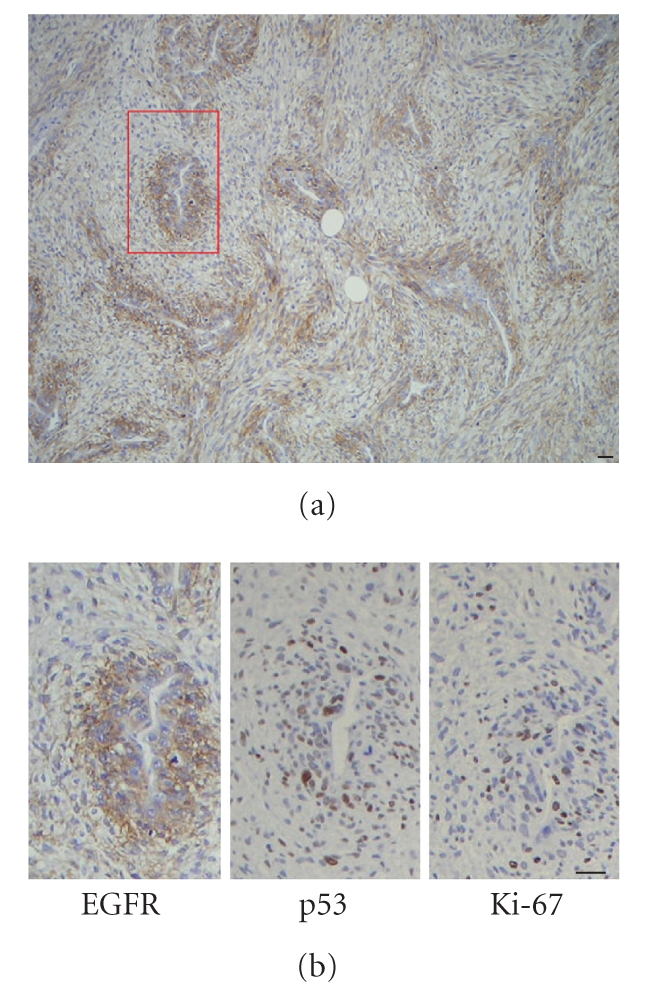
Heterogeneous expression of EGFR (a) and colocalization with high-grade Ki-67 and p53-positive (b) areas. Scale bar represents 15 *μ*m.

**Figure 3:**
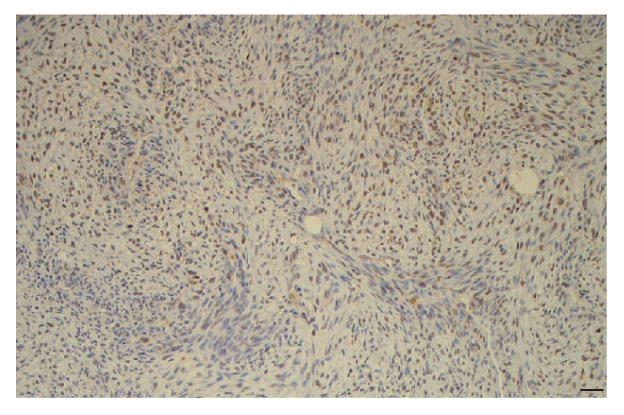
Homogenous expression of survivin in MPNSTs. Scale bar represents 20 *μ*m.

Interestingly, the staining was heterogeneous throughout the tumour in several cases (see Figure 2(a)). In these cases, EGFR-positive cells were localized in “high-grade” areas, defined as areas with high-cellular density and high mitotic index. In four of these cases, samples were available to perform serial sections, and we confirmed that EGFR-positive areas segregate with high-grade features, as proliferative index detected by Ki-67 expression, and in two cases also with P53-positive areas (see Figure 2(b)). By contrast, staining with survivin, which was positive in all the cases of MPNST, was diffuse to all tumour areas (see Figure 3).

To confirm, by another way, the high frequency of EGFR overexpression in MPNSTs, we quantified *EGFR* transcripts in an independent series of 20 MPNSTs using real time RT-PCR, and compared it to 14 benign dermal neurofibromas. The mean of *EGFR* RNA level was higher in MPNSTs than in benign dermal neurofibromas (1.68 ± 2.5 versus 1 ± 0.4, NS), and four (25%) of MPNST samples showed marked increases of *EGFR* transcripts (more than 3 times higher than the mean for benign dermal neurofibromas).

**Figure 4:**
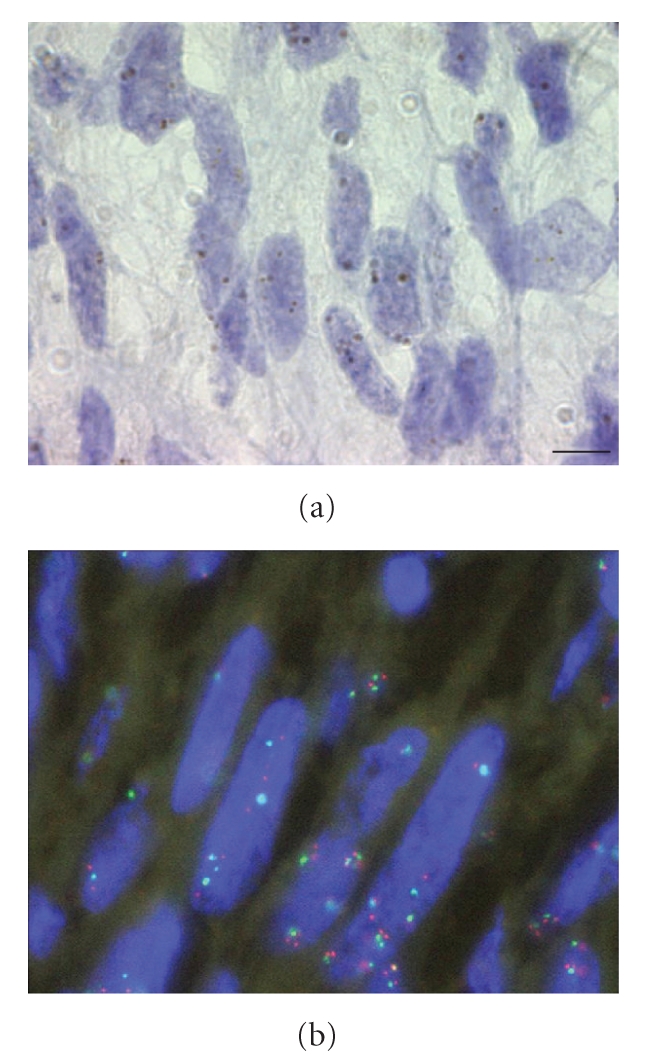
CISH and FISH analysis on the R84 MPNST sample. (a) The mean number of spot detected in the nuclei of 40 to 60 tumour cells in this sample stained by CISH was 3.3. (b) FISH confirmed multiple spot of *EGFR* (red) but revealed a polysomy of chromosome 7 (green) for this tumour. Scale bar represents 5 *μ*m.

To determine whether overexpression of EGFR protein and RNA might be related to an amplification of *EGFR* locus, we performed CISH and FISH analysis on eight MPNST samples of the main series (R72, R74, R78, R84, R85, R86, R92, R94, R111, R116), whose expression was either homogeneous (*n* = 2) or heterogeneous (*n* = 6). None of the tumour had evidence of *EGFR* amplification. The mean number of spot detected in the nuclei of 40 to 60 tumour cells by sample stained by CISH was 2.42 [range from 2.1 to 3.3]. In only one case, 5 to 6 spots were detected in some tumour cells, however FISH revealed a polysomy of chromosome 7 for this tumour (see Figure 4).

All the patients underwent surgical resection of the tumour, except two whose diagnosis was performed at metastatic stage. Two other patients were lost of view, few days after initial diagnosis, and were thus excluded for survival analysis. Kaplan-Meyer analysis of overall survival revealed that the local stage (local or locally advanced) as well as NF1 status had a poor outcome (*P* = .0005 and *P* = .008, resp.), while age at diagnosis, gender, and EGFR expression had not. Multivariate analysis revealed that only NF1 status persisted as an independent prognostic factor (*P* = .02), with a hazard ratio at 2.7 [1.2–6.2]. Median survivals of patients with or without NF1 were 18 and 43 months, respectively, and the 5-year survival was 11% and 45%, respectively.

## 4. DISCUSSION

In this well-defined series of 52 patients with MPNSTs, we have shown by immunohistochemistry that EGFR was expressed in 86% (71–94%) of cases. In the independent series of 20 MPNST RNAs, we observed marked *EGFR* RNA overexpression in 4 (25%) MPNSTs (>3 times the levels in benign dermal neurofibromas). Our results confirm previous detection of EGFR in 8/12 cases by immunohistochemistry [10, 13], in 6/7 patients by western blotting [13], as well as *EGFR* mRNA in 16/42 cases [17]. In the latter study, NF1 patients were more frequently positive for *EGFR* RNA expression (12/25 versus 4/17 in non NF1 patients). Analysis of human MPNST cell lines also revealed a stronger and more diffuse expression of EGFR in cells lines derived from NF1 as compared to non-NF1 patients [18]. EGFR was also more frequently expressed in NF1 patients in our series (95% versus 75%; *P* = .03). Immunohistochemical EGFR detection data has been reported in numerous publications with good staining sensitivity and specificity on paraffin embedded tissue samples.

Overexpression of both protein and RNA suggests pretranslational regulation of EGFR in MPNSTs. Amplification of gene locus is a common mechanism of regulation of EGFR in other tumours such as head and neck squamous cell carcinomas [19], non-small-cell lung carcinomas [20], and colorectal carcinomas [21]. In MPNSTs, *EGFR* amplification has previously been reported in 5 out of 17 patients [22]. In this study, a “low-level” amplification was described with scattered cells containing 6–12 spots, accompanied by polysomy 7 in three cases. Another group failed to detect *EGFR* amplification in four cases, although 1-2 extra copies were seen in one of these cases [23]. Here, we confirm these latter results in eight patients. Thus, EGFR overexpression in the majority of MPNSTs is not due to amplification of the *EGFR* locus at 7p12. Normal Schwann cells do not express EGFR, while NF1 mutation leads to EGFR overexpression in these cells [12]. NF1 loss of function may thus enhance transcription of EGFR.

Mice with heterozygous deletions of *NF1* (*Nf1^+/−^*) do not have an increased incidence of nerve tumours; however when these mice also carry a heterozygous mutations of *TP53* (*Nf1^+/−^*p*53^+/−^*) they develop sarcomas and brain tumours [24, 25]. EGFR is frequently expressed in Schwann cell lines derived from these (*Nf1^+/−^*p*53^+/−^*) mice. Cell growth in these lines is greatly stimulated by EGF and blocked by EGFR antagonists [11]. Decreased EGFR signaling in *Nf1^+/−^*p*53^+/−^* mice reduced their mortality [12]. In the present series, we showed that high expression of EGFR was present in the high-grade areas of the tumours, appearing to colocalize with Ki-67 in all cases and with p53 in half of the cases. In several cases, a strong EGFR expression of in highly cellular regions, contrasting with negativity in other regions, has already been described in one NF1 patient [10]. Thus, as for animal and in vitro models, our data suggest that EGFR overexpression is associated with malignant transformation of Schwann cells.

However, NF1 status was the only prognostic factor in multivariate analysis, with median survivals of 18 and 43 months for patients with or without NF1. EGFR expression, although higher in NF1 patients, did not appear as a prognostic factor for MPNST, nor did local stage, age at diagnosis, or gender.

Overexpression of *survivin* mRNA in MPNSTs has been observed independently by three groups [16, 17, 26]. Supervised analysis of gene expression profiling of MPNSTs revealed that *EGFR*-positive and -negative tumours had a specific gene expression signatures [17]. Interestingly, these authors showed that *EGFR*-positive tumours had a higher expression of *Ki-67* and *survivin* transcripts. We confirmed herein by immunohistochemistry, that bring supplementary data about cellular localization of the expression, that survivin was expressed by malignant Schwann cells. But, contrasting with EGFR, survivin expression was not restricted to high-grade areas of the tumours.

The prognosis of MPNSTs is poor, with only 23% of living individuals 10 years after diagnosis [27]. Post-surgical irradiation, as no effect on overall survival and no effective chemotherapeutic regimens, is available [1]. In our series, the mean age of diagnosis was 23 ± 15 years and the median survival was 30 months. Thus, there is considerable interest in establishing the mechanisms responsible for MPNST tumourigenesis and using this information to develop new, more effective, therapies. Targeted therapies using monoclonal antibodies against EGFR are highly effective in several human cancer [28]. So far, most of the patients treated with a monoclonal antibody anti-EGFR Cetuximab (Erbitux, Lyon, France) suffer from colorectal [29] or lung [30] cancers. Recently, Cetuximab was successfully used to prevent the development of neurofibromas in a mouse model of NF1 [31]. Several groups showed the implication of *EGFR* expression in malignant transformation of Schwann cells in cell lines and/or mouse models. Our results obtained in a large series of human MPNSTs confirmed these data. Tumours with tyrosine kinase receptor overexpression have been successfully treated with targeted therapies, as in gastrointestinal stromal tumours, which express KIT and may be treated with Imatinib [32]. Lung adenocarcinomas, in which the expression of EGFR has no prognostic value [33], may also be treated with gefitinib or erlotinib. Thus, the overexpression of EGFR in 95% of NF1 patients with MPNST and the very poor prognosis of these young patients shown in the present study suggest that new therapies targeting EGFR might be interesting for these patients.

## Figures and Tables

**Table 1:** *Characteristics of the 52 patients with MPNST*. NF1: Neurofibromatosis type 1; Spo: sporadic form of NF1 (no familial history), Fam: familial form of NF1; L: local stage; LA: locally advanced stage; Met: metastatic stage. A: alive. D: dead; +: positive staining; −: negative staining; n.e.: not evaluable staining.

Patient *n*°	NF1	Age/gender	Stage init	Localization	EGFR
R72	no	37/m	LA	Neck	+
R69	no	12/m	LA	Thigh	+
R92	no	11/w	LA	Brachial plexus	+
R78	no	28/w	L	Left arm	+
R94	no	52/m	L	Ethmoid	+
R86	no	33/f	L	Arm	+
R87	no	3/f	L	Tibial nerve	+
R77	no	71/f	L	Median nerve	+
R84	no	19/m	LA	Pelvis	+
R90	no	47/m	L	Right thigh	+
R74	no	16/f	L	Foot	+
R85	no	48/f	L	Forearm	+
R93	no	7/f	L	Mandible	+
R79	no	30/m	L	Wrist	+
R89	no	18/f	LA	Infratemporal fossa	+
R88	no	26/m	L	Left calf	0
R75	no	50/f	LA	Retroperitoneum	0
R76	no	23/m	L	Frontal region	0
B1027	no	15/m	LA	Retroperitoneum, pelvis	0
R73	no	7/m	LA	Neck	0
B1499	no	60/m	LA	Mediastinum	n.e.
R80	no	14/f	L	Left orbit	n.e.
B1044	no	7/m	L	Calf	n.e.
R95	no	20/m	Met	Hand	n.e.
R81	no	46/m	L	Forearm	n.e.
B1463	no	19/m	LA	Armpit	n.e.
R123	Fam	15/m	L	Median nerve	+
R118 B	Fam	11/f	LA	Brachial plexus	+
B1357	Spo	16/m	LA	Sciatic nerve	+
R107	Spo	33/m	L	Neck	+
R111	Spo	13/f	LA	Thigh	+
B1064	Spo	44/f	L	Thigh	+
R116	Spo	10/m	LA	Arm	+
R98	Spo	23/f	L	Calf	+
B2387	Spo	29/m	LA	Supraclavicular region	+
R122	Fam	19/f	LA	Groin	+
R104	Spo	25/m	L	Left calf	+
R97	Spo	17/m	LA	Left iliac	+
B1400	Fam	20/m	LA	Chest wall	+
R105	Spo	13/m	LA	Abdominal wall	+
R96	Spo	23/m	LA	Sciatic nerve buttock	+
R102	Spo	23/m	Méta	Retroperitoneum	+
R101	Spo	26/m	LA	Retroperitoneum	+
B1399	Spo	5/m	LA	Urinary bladder	+
R114	Spo	7/m	LA	Thigh	+
B1148	Fam	17/f	L	Thigh	+
bloc t	Spo	32/f	L	Thigh	+
B1793	Fam	32/f	L	Thigh	0
R127	Fam	11/f	LA	Left thigh (sciatic)	n.e.
R119	Fam	10/m	LA	Retroperitoneum	n.e.
R125	Fam	11/f	LA	Upper	n.e.
B1599	Spo	20/f	LA	Pelvis	n.e.
